# National Effort to Re-Establish Heavy Ion Cancer Therapy in the United States

**DOI:** 10.3389/fonc.2022.880712

**Published:** 2022-06-14

**Authors:** Arnold Pompos, Robert L. Foote, Albert C. Koong, Quynh Thu Le, Radhe Mohan, Harald Paganetti, Hak Choy

**Affiliations:** ^1^ Department of Radiation Oncology, University of Texas (UT) Southwestern Medical Center, Dallas, TX, United States; ^2^ Department of Radiation Oncology, Mayo Clinic, Rochester, MN, United States; ^3^ Department of Radiation Oncology, University of Texas MD Anderson Cancer Center, Houston, TX, United States; ^4^ Department of Radiation Oncology, Stanford University School of Medicine, Stanford, CA, United States; ^5^ Department of Radiation Physics, University of Texas MD Anderson Cancer Center, Houston, TX, United States; ^6^ Department of Radiation Oncology, Harvard Medical School and Massachusetts General Hospital, Boston, MA, United States

**Keywords:** heavy ion, cancer therapy, carbon ion, radiotherapy, particle radiotherapy

## Abstract

In this review, we attempt to make a case for the establishment of a limited number of heavy ion cancer research and treatment facilities in the United States. Based on the basic physics and biology research, conducted largely in Japan and Germany, and early phase clinical trials involving a relatively small number of patients, we believe that heavy ions have a considerably greater potential to enhance the therapeutic ratio for many cancer types compared to conventional X-ray and proton radiotherapy. Moreover, with ongoing technological developments and with research in physical, biological, immunological, and clinical aspects, it is quite plausible that cost effectiveness of radiotherapy with heavier ions can be substantially improved.

## Introduction

The purpose of this article is to heighten awareness within the oncology research and therapy community that major academic medical centers in the United States find the need to re-establish heavy ion cancer therapy in the United States. We want to emphasize that bringing heavy ion cancer therapy back to the United States is a national effort, and not just a wild idea of a few individuals. We provide this article to summarize the rationale to support the re-establishment effort and to publish our ideas and recommendations for how this should be accomplished.

More than 60% of all cancer patients in the United States (U.S.) are treated with one or more courses of radiotherapy during the continuum of their cancer care (1–3). Despite their physical and biological limitations, most radiation treatments worldwide are delivered with high energy X-rays generated either by linear electron accelerators or radioactive sources. The physical advantages of dose delivery with protons started its application to radiotherapy in 1954 in the U.S. at Lawrence Berkeley National Laboratory (LBNL), and to date a relatively small percentage of cancer patients globally (<1%) receive proton radiotherapy (4–6).

Heavy ion therapy is a unique form of radiotherapy for the treatment of cancer. It deposits ionizing radiation in cancer cells *via* accelerated charged particles that are heavier than protons. First, a narrow beam of heavy ions is accelerated in a particle accelerator to give enough energy to the ions to penetrate to the depth of the cancer within the human body and kill cancer cells. In general, the speed of the ion correlates with the energy it carries. For example, a speed of approximately 2/3 to ¾ the speed of light is needed for the ions to achieve the energy needed to reach a deeply located tumor (30 cm) in a large patient. After the ions reach the desired speed and energy, they are extracted from the accelerator and are magnetically guided to patient treatment rooms. The ions leave the magnetic transport system and are focused on cancers. They enter through the patient’s skin and travel to the cancer to deposit the therapeutic dose of ionizing radiation. The narrow incident beam is magnetically scanned, and its energy changed to effectively form a broad beam to “paint” the entire volume of the tumor target with ionizing radiation. Typically, multiple such broad beams, incident from different directions, are used to lower the dose outside the tumor and to optimally spare the surrounding normal tissues.

## What Are the Advantages of Heavy Ion Therapy?

Like protons, heavy ions have a finite range in tissue. The speed of heavy ions is tuned in the accelerator in such a way that when they reach the cancer, they deposit most of their initial energy within the cancer, i.e., the ions abruptly stop within the cancer and only a minor, often clinically insignificant energy spills beyond the cancer target. The heavier the ion is, the more significant this spillage would be due to its fragmentation, thereby limiting therapeutic benefit to ions no heavier than oxygen. High energy X-rays, used conventionally to treat solid cancers, do not stop; they continue to travel and deposit energy to healthy tissue beyond the cancer until they exit the patient’s body. The maximum energy deposition for X-rays occurs when they enter the body upstream of the cancer in healthy tissues.

Unlike X-rays or protons, heavy ions do not significantly scatter sideways in tissue, i.e., they have a sharper lateral penumbra, meaning the dose fall-off in the lateral direction perpendicular to the direction of the heavy ion beam outside the treatment volume is very rapid. This allows the therapeutic heavy ion beam to pass more safely closer to healthy organs on their way to cancers than protons or X-rays could. This property can be exploited in using heavy ions to treat cancers that are closer to organs at risk and would otherwise be too risky to treat with conventional X-ray or proton radiotherapy.

Biologically, heavy ions cause a different type of damage to the cancer DNA and microenvironment than protons or X-rays. The reason for this is that they deposit a very large amount (~100 eV) of densely ionizing energy (high linear energy transfer, LET) on the DNA strand scale (nanometers) near the end of their range in tissue. Because of this, heavy ions cause primarily clusters of double-stranded DNA breaks which are more difficult for cancer cells to repair (7, 8) making these ions biologically more effective (higher relative biological effectiveness, RBE), especially in malignancies that are resistant to traditional X-ray and proton radiotherapy, such as hypoxic tumors (i.e., tumors lacking oxygen which are typically resistant to protons and X-rays (9–12) or tumor initiating cells (13–15). In the region upstream of the cancer where healthy cells are present, heavy ions deposit significantly lower sparsely ionizing energy (low LET) than in the tumor, generating simpler and more readily repairable DNA damage like X-rays and protons.

The biological effectiveness of X-rays in the cancer is much lower than that of heavy ions and since the physical properties of X-rays do not allow for sparing of normal tissues as mentioned above, the therapeutic benefit is much lower than heavy ions. For protons, their biological effectiveness in the tumor is like that of X-rays, except for a small region near the end of their range where their RBE also increases due to an increased LET (16, 17). If using conventional fractionation heavy ion therapy, a variable RBE can be applied to make the RBE-weighted dose the same as an X-ray prescription, delivering less dose for the same biologic effect. However, hypofractionation is feasible and preferred for heavy ions and may, along with their lower OER, be responsible for the superior effectiveness for radiation-resistant tumors.

To achieve the same cancer cell kill, one needs to deposit less energy with heavy ions than with protons or X-rays, leading to significant sparing of healthy tissues, especially distal and lateral to the tumor. The exact amount of increased cancer cell kill (9, 11, 18–21) and healthy tissue sparing depends on the cancer and normal tissue type, type of ion, treatment factors and other parameters. These are subjects of intense research (22–25). Since standard fractionation (25-40 treatments, 1.8-2.1 Gy per fraction) of X-ray or proton radiotherapy in many cancers is designed to allow for normal tissue repair between each treatment to mitigate late toxicity, sparing normal tissues with heavy ions would allow for larger doses of radiotherapy to be given with each fraction, leading to fewer treatment days overall, a regimen called hypofractionation. This increases the convenience and lowers costs for the patient. This type of radiotherapy with heavy ions has been demonstrated to be safe and effective (26, 27).

Heavy ions seem to present immunogenic effects such as an increased production of tumor associated antigens and antitumor effects, which may result in reduced ability to metastasize or recur (28–36). The heavier the ion, the more pronounced these effects seem to be (37). Mouse studies (38) also indicate that combining heavy ion radiotherapy with immunotherapy may lead to a novel and effective route of treating cancer, creating a hope for patients who have distant metastases present. Such combination of therapies may be amongst the most effective forms of cancer therapy (39, 40).

The effectiveness of immunotherapy depends on the health of the immune system. It is well known that conventional radiotherapy with photons can severely damage the immune system, which can lead to adverse clinical outcomes. Recent findings indicate that proton therapy, due to the compact nature of its dose distribution patterns, can significantly spare the immune system, and improve survival (41–46). Radiotherapy with heavier ions is likely to be significantly more sparing of the immune system compared to protons due to the fact that heavy ion dose distributions are even more compact and their clinical application employs hypofractionated regimens.

## Heavy Ion Therapy Started in the U.S. in 1973 at Lawrence Berkeley National Accelerator Laboratory (LBNL) 

At this nuclear physics research facility, the Bevalac accelerator was used in 1973 to treat the first patient with neon nuclei and in 1977 with carbon ions (47, 48). Between 1977 and 1992 multiple early phase clinical trials were conducted with these ions and helium. These initial treatments suffered from numerous limitations at the time, including inadequate imaging, inadequate treatment dose calculation algorithms, lack of biological modeling, and unreliable access to beam in facilities intended for physics research. Anatomic imaging (primarily CT scanning at the time) was in its infancy and was not adequately visualizing cancer targets; dose calculation algorithms were simple without accounting for tissue heterogeneity and organ motion and no biological effectiveness modeling was used to guide treatment planning. The ion accelerator and beam delivery technology were laboratory research grade and not reliable for routine clinical patient care. Two thousand fifty-four patients were treated with helium ion beams and 433 with neon ion beams. The patients who received helium ions presented with primary skull base tumors, such as chondrosarcomas, chordomas, meningiomas and ocular melanomas. Patients treated between 1987 and 1992 had better control of their treated tumors compared to those in the earlier period, representing the influence of improved immobilization, treatment planning and delivery, and introduction of MRI for cancer visualization.

## Heavy Ion Therapy Stopped in the U.S. in 1993

In the early 1990’s, this primarily nuclear physics research accelerator was decommissioned making it the last time the U.S. had a therapeutic heavy ion beam for humans. While the U.S. has several high energy accelerator labs, it is challenging to operate a clinical environment in a research laboratory where all the necessary basic clinical infrastructure is missing. Also, therapeutic beam quality and beam reliability are difficult to achieve in non-clinically oriented physics research labs causing therapy interruptions. On days when access to the ion accelerator prevented treatment due to downtime, treatment with low LET radiotherapy, such as X-rays, is often not at or near the same facility. Moreover, in physics research labs the therapeutic program is highly dependent on the research direction of the lab and on the available beam time as these beams are also used for other research projects.

## Heavy Ion Therapy Migrated Outside of the U.S. 

After the closing of the world’s first heavy ion therapy center, three nuclear research facilities around the world adopted heavy ion therapy. In 1994 Japan opened its first carbon ion radiotherapy (CIRT) center, HIMAC, at the National Institute of Radiological Sciences (NIRS) in Chiba, Japan (now QST Hospital). They currently have an active program and have treated the largest number of patients in the world with carbon ions. The German nuclear research center, GSI Helmholtz Centre for Heavy Ion Research (GSI), commissioned a CIRT treatment room in 1997 and China commissioned a CIRT beam line at the Heavy Ion Research Facility in Lanzhou as part of the Institute of Modern Physics of the Chinese Academy of Science. Six more Japanese, three Chinese, one Italian, one Austrian, and two German sites have been built, all as clinical facilities dedicated for patient treatment. Each has a dedicated heavy ion therapy accelerator and multiple patient treatment rooms served by the same accelerator.

The benefit of using heavy ions for cancer treatment was recognized by the government in these countries leading to insurance coverage for the cost of treatment for several cancer types (49–51). Typical examples of insurance coverage include high- and intermediate-risk prostate cancer in Japan, chordomas, chondrosarcomas, osteosarcomas, salivary gland tumors, and tumor re-irradiation in Japan, Italy, Germany, and Austria. In addition, in Austria, other high-risk indications can selectively receive insurance coverage when experts from the appropriate tumor board recommend CIRT due to its potential benefit over X-rays. In the U.S. heavy ion therapy for clinical research and routine clinical care will require FDA clearance of the equipment and possibly clearance for each cancer indication along with the establishment of CPT codes for billing and reimbursement models.

## Heavy Ion Therapy Technology Development Continued After Clinical Heavy Ion Therapy Applications Left the U.S.

Technological development of heavy ion systems continued outside of the U.S. with implementation of significant improvements to the first system. For example, a novel beam delivery method for protons called raster scanning, which uses strong magnets to scan a narrow ion beam across the cancer for dose delivery while modulating the beam energy, was developed in Japan in 1984 (52). GSI developed and implemented raster scanning for CIRT (53). This is unlike the previously used “scattering” method, where the narrow beam was spread out to cover a large cross-sectional area of the cancer by various bulky beam scattering, shaping and compensating devices custom-designed and fabricated for each specific patient. The implementation of scanning beams helped to further conform dose more tightly to the cancer volume.

Both NIRS and GSI have generated their own models to calculate the biological effective dose for carbon ion beams and they have implemented these models in their treatment planning systems (54–59). A German vendor, Siemens, developed the world’s first heavy ion rotating gantry system for ion beam delivery that used conventional, non-superconducting magnets (60). Toshiba Corporation developed a more compact and lighter weight commercially available gantry using super conducting magnet technology which was installed and is in clinical use at QST Hospital (61). Moreover, techniques to mitigate the motion of the tumor and surrounding normal organs (such as breath hold, rescanning, and respiratory gated ion beam deliveries) have also been developed and implemented to treat moving targets with scanning beams (62). These technological advancements have been adopted by commercial vendors (three Japanese and one European) to transform a system that had previously existed as an experimental system in physics research labs into commercial clinical solutions, which are currently in clinical operation at the 14 international centers listed above. Further development is continuing to make heavy ion equipment more compact and affordable.

## Encouraging Preliminary Clinical Outcome Data for Heavy Ion Therapy

Despite widespread proliferation of proton centers worldwide, only a small number of randomized clinical trials comparing proton radiotherapy to conventional X-ray radiotherapy have been completed to define the benefits from the improved proton physical dose distribution (63–65). A search of clinicaltrials.gov on October 27, 2021 revealed 16 phase III and 4 randomized phase II clinical trials for proton therapy including a wide range of malignancies: prostate, oropharynx, early-stage tonsil, early and advanced stage non-small cell lung, esophagus, breast, rectal, liver, unilateral head and neck, glioblastoma multiforme, head and neck, grade II and III glioma, and nasopharynx. NRG Oncology is sponsoring randomized clinical trials for non-small cell lung cancer, hepatocellular carcinoma, glioblastoma multiforme and esophageal carcinoma. Heavy ions in theory will bring additional enhancement of benefits due to their superior physical characteristics and different biological effectiveness. This will need to be confirmed with randomized clinical trials.

At the 14 clinical heavy ion facilities currently in operation worldwide, more than 37,000 patients have been treated with heavy ions; most of these patients received CIRT. The clinical indications are radiation resistant malignancies, such as adenoid cystic carcinoma, mucosal melanoma, non-small cell lung cancer, liver cancer, bone and soft tissue sarcoma; recurrent previously irradiated cancers (especially rectal cancer), advanced gynecological malignancies, esophageal cancer, and pancreatic cancer and hypoxic malignancies. For these, conventional X-ray and proton radiotherapy typically cannot deliver enough therapeutic dose to be effective due to the high risk of severe toxicities of adjacent organs at risk.

Locally advanced unresectable pancreatic cancer is a typical example where CIRT has shown benefit over conventional radiotherapy. The physical and biological characteristics of carbon ions allows for the delivery of larger and more effective doses than X-rays or protons. The Japanese centers conducted a phase II single arm clinical trial in patients with unresectable locally advanced pancreatic cancer where the 2-year overall survival in patients receiving CIRT reached a remarkable rate of 42 percent in comparison to the 12 to 30 percent achievable with conventional radiotherapy (66, 67). Additional data on patients who received higher CIRT dose than the previously published cohort confirmed the promising results with a 2-year survival of ~60% (68). The results of CIRT compared to conventional treatment methods for selected tumors with the focus on overall survival are shown in the **Table 1**. These results were derived from phase I/II clinical trials and were compared to historical controls with all the caveats associated with early phase non-randomized single arm studies; therefore, there is a crucial need for controlled randomized phase III trials to address all the potential biases and confounders or alternate approaches to generate level I evidence (115).

**Table 1:** Comparison of heavy ion therapy with conventional radiotherapy clinical results – examples indicating the potential benefit of heavy ion therapy.

Disease	Conventional	CIRT	Note
Pancreatic Cancer	12-30% (67)	42% (66) & 60% (68)	2y OS, locally advanced, unresectable
Pancreatic Cancer	12-32% (68)	46% (69, 70)	5y OS, pre-op
Rectal Cancer	20-46% (71–76)	50% (77)	5y OS, post-op, recurrent, surgery
Rectal Cancer	3-49% (78–81)	50% (77),	5y OS, post-op, recurrent, chemo+RT
Sacral Chordoma	52% (surgery) (82–84),	74% (82–84),	10y OS, surgery badly impacts QoL
Sacral Chordoma	62% (surgery+X-ray) (82–84),	74% (82–84),	10y OS
Osteosarcoma	10-14% (85, 86)	33-47% (87–90)	5y OS, inoperable
H&N Mucosal Melanoma	35% (+/-surgery, +/- chemo) (91–93)	54% w/chemo (94–97)	5y OS, see (91–97) for more results
Chordoma	15-54% (98, 99)	80% (100)	10y LC, skull base
H&N adenoid cystic carcinoma	27-56% & 24-59% (101, 102)	74% & 68% (101, 102)	5y LC & 5y OS, mostly inoperable, see (97, 101–110) for more results
H&N bone and soft tissue sarcoma	21-58% (108–110)	80% (68)	5y LC, Bone & Soft Tissue Sarcoma
Liver Cancer	Protons 50% & 39% (111)	~70% & 45% (111)	3y OS & 5y OS, HCC, less acute toxicities
Prostate Cancer	70-78% (112, 113)	>80% (114)	5y OS, biochemical free, high risk, G2 or higher urinary and rectal toxicities 4.1% and 0.4% for CIRT. Higher for other modalities.

Y, year; chemo, chemotherapy; OS, Overall Survival; H&N, Head and Neck; LC, Local Control; HCC, Hepatocellular carcinoma; G2, grade 2; CIRT, carbon ion radiotherapy; RT, radiotherapy; QoL, quality of life.

## Heavy Ion Therapy Technology Design and Capabilities Are Continuously Improving

With the ongoing construction and commissioning of the present heavy ion clinical centers, technology innovation continues. The systems that are under construction continue to benefit from the latest technological advancements. For example, a CIRT facility in Yamagata prefecture in Japan and one under construction at Yonsei University in Seoul have the latest superconducting rotating gantry that is 2/3 the size of the gantry in QST Hospital, Japan. This is due to a more compact novel design of scanning magnets that are 1/3 smaller than the existing ones at QST Hospital and an increase of the magnetic field strength in the superconducting magnets (116).

The next generation of heavy ion systems will feature even more utilization of superconductivity to further reduce system size which should translate into cost reduction. Operational cost will decrease with further automation of the accelerator control system. It will utilize high power ion sources that can provide an order of magnitude higher dose rate, which is crucial for large dose hypofractionated treatments. Fast magnetic field changing transport magnets should be installed in the entire heavy ion accelerator and beam transport system. Together with appropriate accelerator control system software modifications, such changes will enable ultrafast (on the order of 1 minute) switching times between various ion types which is crucial for personalized optimization of treatment plans based on biological effect capabilities (117). Novel, fast particle detectors utilized in control systems will enable very precise particle control and highly accurate dose delivery even at the highest dose rates (118). Novel imaging methods will enable volumetric imaging at the isocenter, highly precise range verification, and uncertainty reduction leading to better utilization of the sharp Bragg peaks of heavy ions. Fast and precise dose calculation engines coupled with deep learning algorithms in treatment planning will enable efficient adaptive re-planning, a necessary tool to implement heavy ion treatment.

For CIRT, two biological effect models have been used, the Japanese microdosimetric kinetic model (MKM) and the German local effect model (LEM) (54–59). A commercially available treatment planning system for CIRT is available that uses both models (from RaySearch Laboratories AB, Stockholm, Sweden). Undoubtedly, with further research and experience, these models will continue to improve, and new models will be developed within the U.S. The largest number of patients with the longest follow up treated on phase I/II clinical trials have been treated with the MKM model. Gantry-less systems for upright patient positioning (standing or sitting in a chair) for imaging and treatment delivery are under development and will result in substantial equipment cost reductions.

While it is true that a future system bearing these novel features at low cost is an attractive idea worth research and development, we first need to establish the clinical role and efficacy of heavy ions in patients. Therefore, we argue that current commercially available heavy ion solutions are adequate for today’s needs and if the first U.S. heavy ion centers are constructed now, novel clinical trials can immediately begin investigating the role of heavy ions in U.S. cancer care. Such a center could be operated more cost effectively if most of the treatments are hypofractionated.

## The U.S. Is Contributing to Research Efforts to Improve Heavy Ion Therapy Technology

Heavy ion therapy has not been re-established in the U.S. due to the capital cost of equipment, operational costs, lack of FDA cleared equipment and lack of CPT billing codes and a reimbursement model. Despite its lack of a therapeutic heavy ion center, the U.S. has significantly contributed towards improving understanding of the biological effects of heavy ions and the heavy ion technology for medical use. In 2013, the Department of Energy (DOE), along with the National Cancer Institute (NCI) hosted a Community Input Workshop on Ion Beam Therapy (119). The workshop identified clinical applications and radiobiological requirements for the use of heavy ions in cancer therapy, assessed beam requirements and delivery systems for future heavy ion beam facilities, and identified research and development activities needed to bridge the gap between the current capabilities and future requirements that are needed to maximize the medical benefit of the heavy ion accelerator and delivery systems.

In 2015 the DOE launched the Accelerator Stewardship program to make particle accelerator technology widely available to science and industry by supporting use-inspired basic research in accelerator science and technology, including medical applications for advanced cancer therapies (120). From 2015 to 2017 there were a total of 23 awards, among which four were dedicated to improving heavy ion therapy.

The NCI also awarded several research grants aimed at high LET and heavy ion technology development. These included a grant investigating On-line PET Based Intra-Beam Range Verification and Delivery Optimization for Improved Particle Radiation Therapy and investigating Large-Area Plasma Panel Detectors for Particle Beam Radiation Therapy. In early 2020, the NCI announced a request for applications to investigate “Radiobiology of High Linear Energy Transfer (High LET) Exposure in Cancer Treatment” and awarded three to five grants. The private industry sector has also invested in developing an advanced cancer therapy accelerator (121).

## Cancer Patients in the U.S. Need Access to Clinical Heavy Ion Therapy Systems for Clinical Research

In April 2016, the NCI hosted a multidisciplinary workshop on the biology of charged particle therapy where internationally recognized experts gathered to discuss the current knowledge of particle therapy in cancer acknowledging the limitations of the research data generated so far to show biological benefits of heavy ions and identifying knowledge gaps that need to be filled with ongoing or future research (122). Therefore, we need to continue conducting basic science investigations to uncover the full potential of heavy ions.

However, we would like to stress that the need for continuing basic science research should not be the primary reason for establishing heavy ion centers in the U.S. Presently, options, though limited, do exist to secure heavy ion beam time to perform scientific experiments either in the U.S (123–125) or internationally (126–131). However, it is neither convenient nor cost effective for U.S. investigators to travel abroad and ship biological samples to conduct experiments. Although these convenience factors do hamper active investigations in heavy ions, limited research activities are being performed. The NCI is actively guiding heavy ion biology researchers to collect relevant global data that can be compared across centers and countries for generation of consensus statements and reporting guidelines. In 2017, the NCI commissioned a panel of internationally renowned experts to review the current state of heavy ion research and to generate guidelines for reporting the physical parameters of the particle beams for biological research and future clinical applications.

Although clinical data has been collected on more than 37,000 patients worldwide suggesting potential clinical benefit for heavy ion therapy over standard X-ray radiotherapy for selected cancers (26, 27, 132) none of these patients were treated on randomized clinical trials. Therefore, we need to design prospective randomized clinical trials using the current knowledge and the latest technologies available for radiotherapy to definitively establish level 1 evidence of benefit. We need to treat all patients whose cancer is suitable for heavy ion treatment on novel clinical trials to uncover the full benefit of this therapy. It is challenging and not feasible for U.S. physicians to design and conduct heavy ion related clinical trials at non-U.S. heavy ion centers. In addition to the coordination of logistics, the cost of travel, food and housing, treatment, and follow-up at foreign sites can be substantial.

The NCI has mobilized the international community and funded a dose escalation phase I trial “A Prospective Phase I Clinical Trial of Carbon Ion Radiation Therapy for Locally Advanced, Unresectable Pancreatic Cancer” which irradiates Chinese patients at the Chinese Proton and Heavy ion center in Shanghai (133). To date, no U.S. citizen has been treated on a phase III clinical trial using heavy ion treatments abroad. Only 15 U.S. citizens have been able to travel abroad for CIRT (manuscript under review). Global pandemics have made international travel even more restrictive.

Despite the clear need to construct heavy ion clinical treatment centers in the U.S., there are fundamental practical challenges to overcome in the process. These include the lack of FDA clearance and the lack of a heavy ion therapy reimbursement model in the U.S. The lack of FDA clearance and a reimbursement model will make it impossible to recover the facility initial capital investment and ongoing operating costs. Without a realistic reimbursement model, health care organizations will not be able to create a viable business plan without substantial governmental or philanthropic support. The price of the heavy ion equipment at the first U.S. facility is at the mercy of international vendors and is strongly influenced by the global political and economic climate. The U.S. has no commercial vendor to offer a solution.

## The U.S. Scientific and Medical Communities Are Anxious to Re-Establish Heavy Ion Therapy in the U.S.

In 2015, the NCI awarded two exploratory planning grants entitled “Planning for a National Center for Particle Beam Radiation Therapy Research” (134, 135). These grants were aimed at assisting U.S. institutions that have been actively working to establish heavy ion therapy centers in planning the research component of such a center. A two-day heavy ion dedicated annual symposium named the “International Symposium on Heavy Ion Therapy (ISIT)” started in 2014 to gather U.S. and international heavy ion experts to discuss topics that are relevant in restoring clinical heavy ion therapy in the U.S (136). The symposium also served as a forum to present scientific results to help guide future research at the proposed centers. Moreover, the symposium provides the opportunity for heavy ion therapy vendors to present the latest advancements in their system development to help inform the U.S. institutions on the cutting-edge capabilities of heavy ion systems offered on the market.

Every third year, the Particle Therapy Cooperative Oncology Group (PTCOG) holds its annual meeting in the U.S. The education section provides a valuable opportunity for U.S. institutions to learn about heavy ion therapy benefits and the details of system design and setup while the scientific section is dedicated to presenting the latest basic science and clinical research results from the world experts on particle therapy and to stimulate research interest in the U.S (6). In addition to the staunch effort of the U.S. scientific community to bring heavy ion therapy technology back to this country for cancer treatment, the U.S. congress has also expressed its support. Congress has issued a statement on the Departments of Labor, Health, and Human Services Section of the Omnibus Bill that encourages the NIH to explore further the development of a state-of-the-art heavy ion research facility in the U.S. Furthermore, the congressional statement encourages the NIH to work with the Departments of Defense and Energy, and other applicable Federal agencies to equip the first U.S. heavy ion research center (137).

Mayo Clinic in Jacksonville, Florida has announced plans to build the first clinical/medical grade heavy ion therapy facility in the U.S./Americas. This facility will include a hybrid carbon ion and proton particle accelerator (synchrotron) which will be fully capable of accelerating other ions (such as helium). There will be two proton treatment rooms with gantries and one carbon ion room with a single fixed horizontal port. Groundbreaking is anticipated in the spring of 2022 with protons available for commissioning in late 2025 and carbon ions available for commissioning in 2026.

## Recommendations for Successful Re-Introduction of Heavy Ion Therapy in the U.S.

The following recommendations are based on the reports of workshops and discussions at conferences on heavy ion cancer therapy including some organized by the authors (AP and HC) and planning efforts supported by the Department of Health and Human Services to plan for heavy ion cancer therapy research (119, 122, 135, 136).

We note that there are ample clinical and scientific data confirming that heavy ion treatments are safe, effective, and can be beneficial in several cancer types.

Therefore, we propose the following:

1) The establishment of the first heavy ion therapy facilities in the U.S. should follow a dramatically different path from that of proton radiotherapy.
a. We propose to build a limited number (2–3) of modest sized (1 or 2 treatment rooms capable of treating 300 to 600 patients per year) heavy ion centers, with capacity to accept the appropriate number of patients needed for rapid accrual into clinical trials but small enough to minimize the cost of construction, equipment, and operation. Using the SEER database cancer statistics and indications for heavy ion therapy used in Japan and Germany, the Mayo Clinic in Rochester, MN has estimated that there are approximately 44,340 patients diagnosed with cancers with indications for heavy ion therapy each year in the U.S. The Mayo Clinic in Rochester, MN treats approximately 1200 patients each year with proton therapy. In reviewing these patients, it is estimated that 12-13% would be eligible for heavy ion therapy. Therefore, a limited number of heavy ion therapy facilities in the U.S. would have negligible impact on the existing and planned proton facilities. A review of the Mayo Clinic Rochester Cancer Registry revealed that over 780 patients are evaluated each year who have indications for heavy ion therapy. Practically, the first heavy ion facilities will be developed sequentially rather than simultaneously. To accelerate the completion of phase III clinical trials for each indication for heavy ion therapy, near simultaneous development would be ideal to accrue patients to clinical trials expeditiously. Depending on the funding available, some of the centers may be gantry-less configurations with horizontal beams with upright (standing or sitting) patient positioning for basic research as well as clinical trials for an appropriate subset of disease sites.
b. We hypothesize that the differential RBE and immunomodulatory effects of heavy ions in the cancer and healthy tissue sets heavy ions apart from proton radiotherapy. Hypofractionation should be the approach to test and implement heavy ion treatment clinically. If hypofractionation proves to be the most efficacious approach to deliver heavy ions, as appears to be the case based on past and ongoing trials in Japan and Europe, new reimbursement models that consider the entire course of treatment rather than per fraction reimbursement can be developed. This will help to keep the cost of a course of CIRT in line with a conventionally fractioned course of intensity modulated radiotherapy or proton radiotherapy. It will also allow for higher patient throughput. More importantly, the patients will complete their radiation treatment faster, return to work sooner, and require less support from their family, friends, and employers. Less acute toxicity also means requiring less costly supportive care and an increased ability to receive more systemic therapy if needed for the cancer treatment. Less acute and late toxicity also translates into better function and improved quality of life with lower costs associated with treating adverse events. Improved cancer control will also translate into fewer costs associated with treating recurrent cancer. The cost savings will be quantified in prospective clinical trials.
c. We propose that the first U.S. heavy ion facilities be supported for an initial period by Federal and State agencies, possibly in partnership with commercial vendors and institutions to conduct clinical trials to define the comparative effectiveness and cost effectiveness of heavy ions until the relevant regulatory approval and health care service reimbursement models are established and implemented to help sustain the operation of the centers.
2) The ideal heavy ion treatment facility would have existing patient volumes to match the capacity of the facility, allow easy, rapid and efficient access *via* responsive, respected radiation oncologists with particle therapy and subspecialty expertise who will communicate openly and in a timely manner with referring physicians, patients and their families; access to photon and proton therapy on site to ease randomization, radiation therapists, certified medical dosimetrists and medical physicists with particle therapy expertise; educational infrastructure to train the next generation of heavy ion therapy providers, research infrastructure to conduct basic science and clinical research with an established track record of clinical trial expertise, and an NCI-designated comprehensive cancer and comprehensive academic medical center to provide surgery, medical therapy, rehabilitative services, social services, psychologic support, chaplain services for spiritual support, and concierge services to help patients and family members with referrals, the intake process, transportation, food, housing, entertainment, and insurance coverage.
3) To continue supporting research and development that will lead to more compact, efficient, and less expensive heavy ion technologies. These will help to make heavy ion treatment accessible to more patients once its benefit is defined by the clinical trials conducted in the initial U.S. centers. However, the initial centers will need to be established with the current state of the art technology to conduct the trials as soon as possible.
a. We expect that the initial U.S. heavy ion therapy systems, as any other new technology in its infancy, need to be safe, effective, and function reliably. Their primary function would be to deliver clinical beam to conduct clinical trials with basic and translational research components.
b. We theorize that if clinical trials prove the benefit of heavy ion therapy in selected patients, these results, combined with cost-effective treatment strategies and technology advancements, will lead to appropriate proliferation of heavy ion centers driven by reduced cost, increased health care demands, and other market forces. But first, the U.S. needs the establishment of a limited number of supported heavy ion therapy centers now.
4) Private, federal, state, and philanthropic funding support are essential to establish, construct, and equip a limited number of heavy ion centers in the U.S. that utilize currently existing technologies in the market.

## Discussion

There is strong basic physics, biology, immunomodulatory and clinical evidence to document the safety, efficacy, and potential benefits of heavy ion radiotherapy to the extent that it must be clinically tested and implemented in the U.S. A limited number of centers in the U.S. should be established and supported now to conduct clinical trials in conjunction with basic and translational research to ascertain the appropriate indications for heavy ion radiotherapy. In the meantime, technological development should continue to develop more compact, efficient, and less expensive sources of therapeutic energy heavy ion beams, ion delivery systems, and dose verification processes. New reimbursement approaches should consider the course of treatment based on the hypofractionation benefit of heavy ions, leading to improved oncologic, quality of life and functional outcomes, and reduced health care costs in the long term. We assert that, even with higher initial investments, the overall value may be equivalent and possibly better than protons and other conventional radiotherapy modalities.

## Author Contributions

All authors (AP, RF, AK, QL, RM, HP, and HC) have made substantial contributions to conception and design (AP, RF, AK, QL, RM, HP, and HC), acquisition of data (AP, RF, AK, QL, RM, HP, and HC), analysis and interpretation of data (AP, RF, AK, QL, RM, HP, and HC); and have been involved in drafting the manuscript or revising it critically for important intellectual content (AP, RF, AK, QL, RM, HP, and HC); and given final approval of the version to be published (AP, RF, AK, QL, RM, HP, and HC). Each author has participated sufficiently in the work to take public responsibility for appropriate portions of the content (AP, RF, AK, QL, RM, HP, and HC); and agreed to be accountable for all aspects of the work in ensuring that questions related to the accuracy or integrity of any part of the work are appropriately investigated and resolved (AP, RF, AK, QL, RM, HP, and HC).

## Conflict of Interest

Author RF was employed by Mayo Clinic.

The remaining authors declare that the research was conducted in the absence of any commercial or financial relationships that could be construed as a potential conflict of interest.

## Publisher’s Note

All claims expressed in this article are solely those of the authors and do not necessarily represent those of their affiliated organizations, or those of the publisher, the editors and the reviewers. Any product that may be evaluated in this article, or claim that may be made by its manufacturer, is not guaranteed or endorsed by the publisher.
